# Integration of Human African Trypanosomiasis Control Activities into Primary Healthcare Services: A Scoping Review

**DOI:** 10.4269/ajtmh.19-0232

**Published:** 2019-09-03

**Authors:** Philippe Mulenga, Faustin Chenge, Marleen Boelaert, Abdon Mukalay, Pascal Lutumba, Crispin Lumbala, Oscar Luboya, Yves Coppieters

**Affiliations:** 1 Faculty of Medicine and School of Public Health, University of Lubumbashi, Lubumbashi, Democratic Republic of the Congo;; 2 Department of Public Health, Institute of Tropical Medicine, Antwerp, Belgium;; 3 School of Public Health, Université Libre de Bruxelles (ULB), Brussels, Belgium;; 4 Centre de Connaissances en Santé en République Démocratique du Congo, Kinshasa, Democratic Republic of the Congo;; 5 Department of Tropical Medicine, Faculty of Medicine, University of Kinshasa, Kinshasa, Democratic Republic of the Congo;; 6 National Program for the Control of Human African Trypanosomiasis, Kinshasa, Democratic Republic of the Congo

## Abstract

Human African trypanosomiasis (HAT) also known as sleeping sickness is targeted for elimination as a public health problem by 2020 and elimination of infection by 2030. Although the number of reported cases is decreasing globally, integration of HAT control activities into primary healthcare services is endorsed to expand surveillance and control. However, this integration process faces several challenges in the field. This literature review analyzes what is known about integrated HAT control to guide the integration process in an era of HAT elimination. We carried out a scoping review by searching PubMed and Google Scholar data bases as well as gray literature documents resulting in 25 documents included for analysis. The main reasons in favor to integrate HAT control were related to coverage, cost, quality of service, or sustainability. There were three categories of factors influencing the integration process: 1) the clinical evolution of HAT, 2) the organization of health services, and 3) the diagnostic and therapeutic tools. There is a consensus that both active and passive approaches to HAT case detection and surveillance need to be combined, in a context-sensitive way. However, apart from some documentation about the constraints faced by local health services, there is little evidence on how this synergy is best achieved.

## INTRODUCTION

Human African trypanosomiasis (HAT), also called sleeping sickness, is a vector-borne disease, which mainly affects poor populations living in rural areas in sub-Saharan Africa. It is transmitted to humans by the bite of an infected tsetse fly. Two parasite species can cause sleeping sickness, *Trypanosoma brucei* (*T.b.*) *gambiense*, and *T.b. rhodesiense.*^1^ The form caused by *T.b. gambiense* is anthroponotic and occurs in 24 countries of sub-Saharan Africa, causing up to 98% of all HAT cases. It is estimated that 57 million of people live in areas at risk for *T.b. gambiense* HAT, the focus of our study.^2,3^ Currently, the main HAT control strategy, case detection and treatment, is organized actively (by a specialized team that moves to the population at risk or passively when a patient seeks care at the level of a health facility).^4^ In practice, and for decades, HAT control in Central and West Africa has been essentially implemented in a “vertical” approach whereby specialized mobile teams screen at-risk populations once a year, and treat confirmed HAT cases in the villages or in referral centers.^5^

In The Democratic Republic of the Congo (DRC), toward the end of the 19th century, there were devastating epidemic of HAT.^6^ At that time, the causative agent of the pathology was not known and no effective treatment was available. By the 1920s, the HAT epidemic persisted over several decades until the 1930s, where the number of cases decreased from 33,502 to 11,837 in the 1940s. This decrease was due to the mandatory screening and treatment performed by mobile units examined in villages that reported outbreaks.^7^ It should also be noted that it was around 1945 that there was the inauguration of the first mass prophylaxis campaign in Kwango, first propamidine, then pentamidine which was better tolerated. These campaigns quickly spread to all endemic areas, and it was reported that about 2,000,000 people regularly received their pentamidine injection every 6 months.^6^ In 1958, 1,218 new cases of about 6 million screened were reported.^7^ All of these efforts throughout the colonial period had reduced the incidence of sleeping sickness on the continent. Vertical programs operated with some degree of integration with the general health services. In colonial times, the local Health Medical District management team organized the periodic annual mass screening of the population. De Brouwere and Pangu called this model “managerial integration.”^8^ This mode of active mass screening of that time was fiercely criticized, when the debate about the integration of vertical disease control program activities into the first-line health services started.^9^ The debate argued about the contrast between the militaristic approach of colonial campaign compared with the patient-centered approach of primary healthcare. In the specific case of sleeping sickness control, most policy makers were pleading for a combination of the two approaches.^10–12^ In the field, in the early years of independence, health services in the affected regions faced serious budgetary and operational constraints, and after 4 years of sustained low endemicity, HAT control was no longer a priority.^5^ The outbreaks reappeared in 1964 after the collapse of the control system in the early 1960s. Control activities resumed in 1964 with technical assistance and funding from Belgium.^7^ However, in 1967, the newly independent Republic of Congo created a sleeping sickness program on presidential decree, which established an entirely vertical program, under strict line management by the director of the program and delivering all services.^8^ The efforts of that time had succeeded in keeping the annual number of cases detected below 7,000 until the mid-1980s. In the following years, a resurgence occurred due to the reduction of active case detection activities. This was due to the expulsion of the Belgian Cooperation for 2 years. This cooperation was the only bilateral donor of the national HAT control program until the 90 seconds. From 1998, an increase in the population screened (from 705,434 in 1993 to 1,472,674 in 1998) and the number of detected cases (from 5,825 in 1991 to 26,318 in 1998) were observed thanks to the strengthening of HAT control.^7^ In DRC, the number of cases continued to decline in the subsequent years from 16,951 in 2000 to 5,968 in 2012.^13^ Since the observed peak in 1998, with 37,385 reported cases of HAT, the number of reported yearly cases in the last 3 years has steadily fallen to less than 3,000,^14^ the lowest level since the data collection started 75 years ago. Globally, the number of reported new cases of HAT was less than 2,000 in 2017 and the DRC was reporting more than 75% of this number. Intense control efforts by the affected countries and the international community over the past 20 years most likely contributed to this decrease. The WHO considers HAT as one of the Neglected Tropical Diseases (NTD) eligible for elimination as a public health problem by 2020. The target for HAT elimination was set as “less than 2,000 cases globally or a reduction by 90% of the total area at risk reporting ≥ 1 case/10,000 people/year.”^15^ A second target was set in 2030, with the objective to stop the transmission of the pathogen to humans by that time. These goals have been endorsed in the London Declaration of 2012 on NTD,^16^ and in this context, “integration of NTD control” is often endorsed.^17^ The changing epidemiological context also calls for different service organization approaches.

The DRC HAT control program (*Programme National de Lutte contre la THA* [PNLTHA]) continues until today to organize the annual active screening campaigns and manages a number of vertical structures, called the *Centre de Diagnostic, Traitement et Contrôle* (CDTC). Many HAT cases are de facto detected in fixed health facilities,^12^ where the patient seeks care, often at an advanced stage. This case detection mode is called passive screening. From all the reported HAT cases in 2015 in DRC, about half were detected during active and half during passive screening. Most of the health facilities implementing “passive screening” were CDTCs, that is, PNLTHA-supported specialized diagnosis and treatment centers. In reality, hardly any first-line primary healthcare centers or rural hospitals contributed to passive screening. The first-line health centers have to cope with unskilled staff, lack of resources, and low attendance rates.^4^

After 10 years of research and development, Drugs for Neglected Diseases initiative (DNDi) and partners developed fexinidazole as the first oral treatment for sleeping sickness. In 2018, this drug received a favorable opinion from the European Medicines Agency for the marketing of this treatment.^18^ The DRC issued on December 24, 2018 a marketing authorization for fexinidazole for the treatment of HAT.^19^ Fexinidazole is the only oral treatment for sleeping sickness that has been shown to be effective in both phases of the disease. This molecule represents a tremendous advance for all countries affected by this tropical disease, especially for people living in the most remote areas and, therefore, far from medical centers able to provide treatment against HAT. Its distribution will make a big step toward the gradual elimination of this disease from sub-Saharan Africa.^18,20^ And in the context of diagnosis, there has been technological improvements such as the development of the HAT-rapid diagnostic test (RDT)^21–23^ and sensitive concentration tests such as the WOO test or the capillary tube centrifugation technique (CTC)^24,25^ and the mini-anion-exchange CTC on buffy coat (mAECT-BC).^26^ First-line health centers are now able to conduct a screening test for HAT in presumptive patients. With the HAT-RDT, the services of sleeping sickness have been radically reorganized in many countries to foster service integration, including in crisis settings.^27,28^ WHO now recommends the integration of surveillance activities in the first-line health services based on the use of RDTs.^2^ Two RDT formats are now available, in a single-use format, to be stored at an ambient temperature and needing a minimum of training.^29^ Because of low specificity levels (around 90%), positive RDT-HAT cases should always be examined by more specific parasitological confirmatory tests.^30,31^ Electricity, equipment, and skills are required to perform these confirmation tests; therefore, it cannot be made available everywhere and this constitutes a big hurdle.^4^

In the specific HAT context, the second disease control strategy is vector control. Integration of tsetse control has less been an issue for debate so far because of lack of appropriate technology. This previous technology was good, but it was expensive for a large-scale deployment. A new approach based on tiny targets is considered promising and might in the future also be looked at from an integrated control perspective.^32^ Nonetheless, as the past “integration” debate has concentrated on how HAT screening and treatment could be provided and managed by regular primary healthcare services, this will the topic of our scoping review.

This scoping review aims to reveal what is known and unknown so far about the integration process of HAT in the first-line health services, the lessons learned as well as the arguments for integration to guide any implementation of the integration of HAT control activities in the era of elimination.

## METHODS

The research question we have defined is “What is known about the integration process of HAT case detection into first-line health services and what are up to now the lessons learned in different settings?”

### Definitions.

The term “integration” is used in different contexts and in different ways in the public health field. Integration of the activities of a given disease control program into mainstream primary healthcare services can, therefore, be defined as follows: “the decision to carry out the activities of a disease control program by staff working in horizontal structures leading to a transfer of responsibility for disease control activities from a specific (or vertical) disease program to general (or horizontal) health service staff.”^33^ A horizontal structure is decentralized and works permanently. The staff of the basic health services is versatile in that it is able to take care of all health problems that the population presents. This notion of versatility is opposed to that of specialization, characteristic of vertical structures.^34^ In the literature on HAT control, the term “integration” generally refers to the transition from a vertical or specialized facility to a horizontally organized structure. In this context, the term “vertical” may apply to a disease-specific program or structure. It is also useful to point out that integration can be administrative, operational, or both. Administrative integration means that the activities carried out by specialized services are now under the control of the general health system and its management apparatus (whereas they were previously under the control of the management of the specialized program). Operational integration implies that specialized control activities are now carried out by staff of the primary healthcare services (whereas they were previously operationalized by the specialized teams).^34^

### Search strategy.

A scoping review aims to describe in quite some narrative detail all research in a particular field to audiences as end users, decision makers, caregivers, or general practitioners. This approach does not contain a formal evaluation of the methodological quality of studies, but it provides a broad overview of research methods and findings dominating the subject. The analysis in a scoping review is based on all the types of studies encountered, independently of the study design.

This scoping review followed the methodological guidance as described by Arksey and O’Malley^35^ in 2005 as well as by Levac, Colquhoun, and O’Brien^36^ in 2010. We have subsequently 1) identified the research question; 2) conducted a literature research; 3) selected the studies; 4) extracted the data; 5) summarized and reported the results; and 6) consulted stakeholders on the results by presenting preliminary findings at the ISCTRC meeting in Zambia in September 2017.

**Figure 1.**
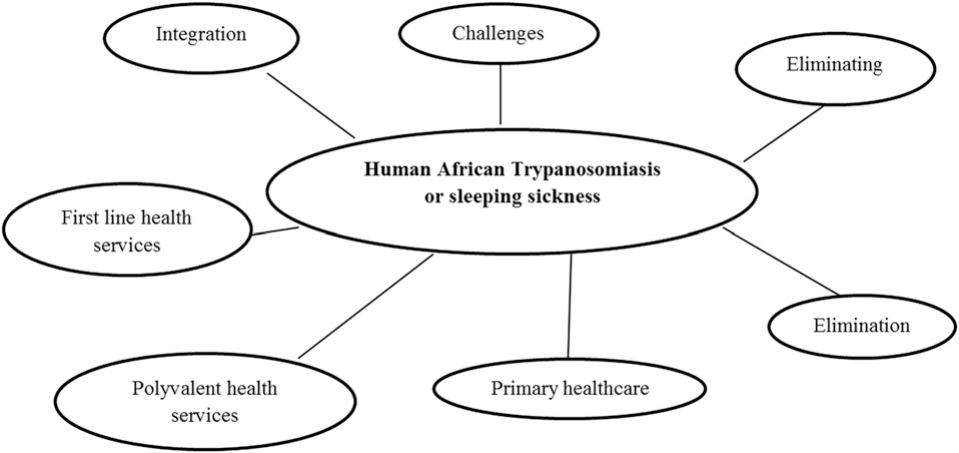
Search strategy.

We searched electronic data bases (PubMed and Google Scholar) as well as data bases containing gray literature, such as the archives of the National HAT Control Program of the DRC and the data bases of the library of the Institute of Tropical Medicine of Antwerp on the integration of HAT. Data bases have been searched for the literature published between 1977 and 2017. The choice of this period corresponds with the moment of debate on the promotion and implementation of primary healthcare with the adoption of the Alma Ata declaration on primary healthcare.^37^ Details on search terms and keywords of Medical Subject Headings (MeSH) are shown in Figure 1.

We included documents meeting the following inclusion criteria in this review: 1) published in English or French, 2) on passive screening for HAT in general or in particular on passive screening in the DRC, 3) on the integration of HAT screening in health facilities in the DRC, and 4) published between 1977 and 2017. The exclusion criteria used to filter the different documents were 1) published in another language than French or English, 2) dealing with the active screening of HAT, 3) dealing with the integration of HAT from another country in a particular way, and 4) published before 1977. We did not restrict on study design nor the type of the article. We also checked the references of all selected documents to identify others meeting our inclusion criteria.

**Figure 2.**
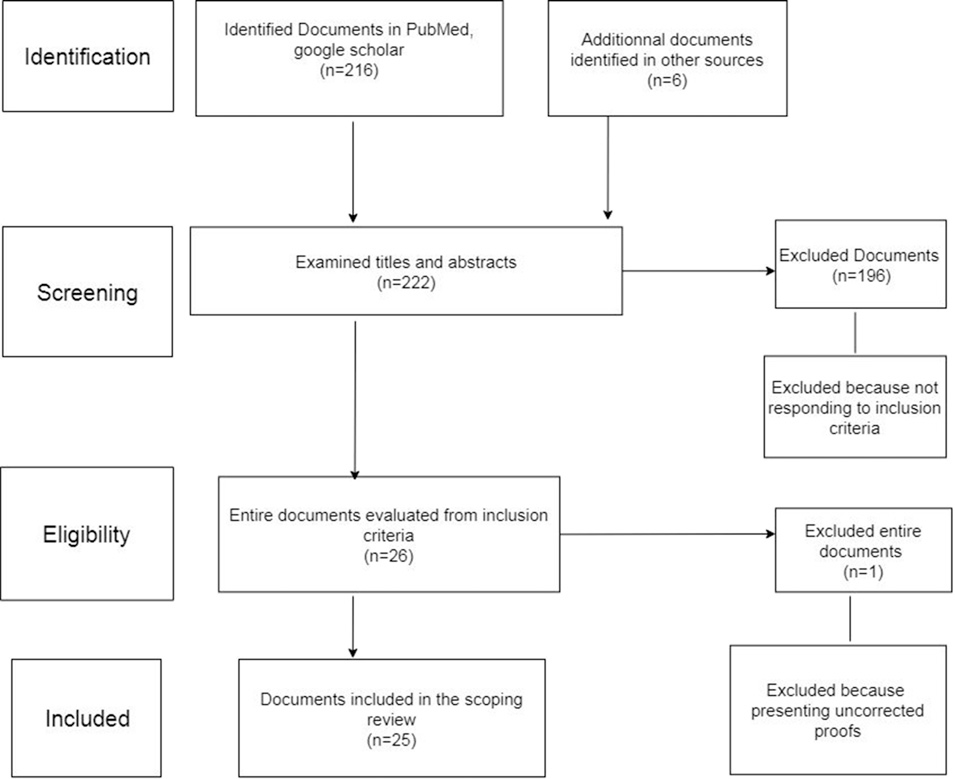
Diagram of the research strategy of literature.

Two researchers developed and conducted this literature search. First, they checked each document using the title and the abstract to discern those meeting the inclusion criteria. For all the abstracts meeting the inclusion criteria, the full documents were retrieved. Next the full text of selected articles was read. Last, the researchers extracted the data of all the included documents. This form contained rubrics allowing to record relevant information on the integration of HAT contained in each document. The diagram of the research strategy of the literature is depicted in Figure 2.

## RESULTS

The research identified 222 documents from PubMed, Google Scholar, and gray literature of which 197 were excluded by the criteria mentioned earlier. Twenty-five documents were included in this scoping review. Most of the documents were published after 1998 (*n* = 20, 80%) (Table 1). The other documents are from the late 1970s and 1980s (Table 2). Nine were original articles, five were reviews, and the others were viewpoints (*n* = 3), debate (*n* = 2), editorial (*n* = 1), PhD thesis (*n* = 1), and policy papers (*n* = 4). We will first describe the main arguments given for the integration of HAT control, then the enabling and inhibiting factors for integrating case detection, the lessons learned, and finally the current policy on integration.

**Table 1:** Overview of documents published or written before 1998 (*n* = 5)

Reference	Study design	Arguments for integrated HAT screening	Favorable factors (F)/Obstacles (O)	Lessons learned
Mercenier^43^	Descriptive case series (*n* = 384 HAT cases)	1. Potential for earlier detection of HAT (this was era before CATT)	**F**: Fixed health centers are permanent and better accessible	63% of all the HAT cases confirmed in the district had consulted spontaneously a health center
Pepin et al.^41^	Case study in Nioki district, DRC	1. Declining uptake of active screening for HAT and better acceptability of integrated screening	**O1**: HAT cases detected in primary health centers are at more advanced stage (only 22% stage one compared with 65% in mobile units)	1. Primary healthcare centers can contribute substantially to HAT screening (within 5 years from start, health centers detected 31% of all HAT cases in the health district)
		2. Lesser cost of integrated screening	–	2. Active screening needs to be maintained and could not be organized by the health center nurse in the case of Nioki
		–	–	3. Essential requirements for integrating HAT screening in the health center are: functional and well-equipped health center, with adequate utilization rate, and trained and motivated staff
Debrouwere and Pangu^8^	Viewpoint	Primary healthcare policy	–	The operational modality of integration has to be carefully chosen depending on context. Integration can be operational, administrative, or complete (both administrative and operational), depending on which responsibilities are handed over to the general healthcare system.
Kuzoe^40^	Review	1. Coverage	**F**: New tools open perspectives for integrated screening	–
Kegels^49^	PhD thesis	–	**O:** A lot of internal and external resistance toward integration leads to a very slow process	–

HAT = Human African trypanosomiasis. (Empty boxes means that the document you are viewing does not contain information related to the title of the column.)

**Table 2:** Overview of documents published or written après 1998 (*n* = 20)

Reference	Study design	Arguments for integrated HAT screening	Favorable factors (F)/Obstacles (O)	Lessons learned
Van Nieuwenhove et al.^7^	Analysis of routine HAT data reported to PNLTHA in DRC 1989–98	–	–	A functional health service can miss a nearby HAT resurgence if no active screening/surveillance is conducted
Louis^9^	Debate	–	–	A combination of active and passive screening, and vector control is required to avoid resurgence
Simarro et al.^10^	Analysis of global reported HAT data 1997–2006	Sustainability	**O**: The existing tools inadequate for primary health center. Lack of a sensitive and specific diagnostic test and of a drug that is cheap, safe, and easy to administer.	“*Sustainability can only be achieved through an integration of activities in a strengthened health system able to face such responsibilities. The current approach should include specialised teams and healthcare systems, rather than falling back on the former debate between the value of specialized teams or primary healthcare. In other words, specialized teams and primary healthcare need to work together synergistically*”
Rapport de Formulation^39^	Policy paper	Cost coverage	–	“*Une stratégie d’intégration est le seul moyen de répondre au fait que les unités mobiles n’arrivent assurer une couverture totale*”
Hasker et al.^45^	Random sample survey on health-seeking behavior	1. Declining uptake of active screening for HAT and better acceptability of integrated screening	**O1**: Very long patient and health system delays (median 4 and 2 months, respectively) in integrated screening before reaching a HAT diagnosis	1. Financial barriers explain long patient delay
			**O2**: High out of pocket expenditure (median 44 USD) before HAT diagnosis is reached	2. Patient delay was less at primary healthcare services (1 month) compared with dedicated CDTCs (4 months) but healthcare system delay was higher (7 vs. 0 months)
			**–**	3. Good synergy between screening at first level and confirmation at specialized center/hospital is required
Tong J et al.^50^	Debate	–	–	In conflict zones, integrated approaches may be not be feasible
Strategic plan PNLTHA^55^	Policy paper	Coverage	**F**: Doctors, nurses, and laboratory staff were trained in endemic zones. Proportion of cases detected in fixed health services rose from 15% to 18% from 2007 to 2009	–
Mitashi et al.^52^	Systematic review	–	**F**: New diagnostic tools open perspectives for integrated screening	CATT and RDT are appropriate formats for HAT screening at first-level PHCs. No appropriate format for HAT confirmation available for this level
Hasker et al.^42^	Viewpoint	Cost sustainability	**O1**: Long delays in PS	“*Gradual and well-managed integration of the HAT control activities in the general health services can be envisaged, but this will require further research to identify the best strategies in low prevalence contexts. A minimal vertical approach to control the disease is likely to remain necessary*.”
			**O2:** More advance stage in PS	
			**O3**: Low attendance rates	
Lejon^48^	Editorial	–	**O1 (related to specificity)**: High degree of cross-reactivity on other antibody detection tests (HIV, malaria) in HAT patients.	–
			**O2 (related to sensitivity**): Reliance on RDTs in PHC centers will lead to missing HAT	
Control and surveillance of HAT WHO^2^	Policy paper	The integration needs of the THA are satisfied by the arrival of the RDT-HAT	**O**: Insecurity, instability	Operational research is recommended to optimize passive screening
Franco et al.^46^	Review	1. Coverage	–	To reach elimination one has to combine three strategies: Active screening, passive screening and vector control, in the right mix, depending on epidemiological and health system context.
				“*Passive screening is the most important component for gambiense HAT control and surveillance in foci with low and very low intensity of transmission, where infection is rare and active case detection has poor cost-effectiveness. Passive screening is also a key element in high, very high and moderate transmission foci, as effectiveness of active screening is limited because of incomplete attendance at screening sessions, inherent limitations of screening tools and the lag between successive surveys*.“
		2. Sustainability		
Franco et al.^3^	Review	Sustainability	**F**: New diagnostic tools open perspectives for integrated screening	-Strengthening health system and increasing population awareness to implement the activities included in the elimination strategies is essential—sustained financial commitment
			**O**: Weak peripheral healthcare system, understaffed and underequipped, with a low coverage or low attendance rate.	
Eperon et al.^53^	Review article	–	**O1**: Current complexity of diagnostic algorithm	–
			**O2**: Current stage two treatment regimen (NECT) is limited to hospital level and not adequate for the first line	
			**O3**: Lack of trained staff and logistic resources in local health services most HAT-affected countries	
Simarro et al.^12^	Capacity mapping survey (fixed health facilities offering DP)	1. Enlarging physical coverage of screening through the network of fixed health centers	**F**: Knowledge of the disease by health staff and affected communities.	1. More than 80% of the population at risk for *Trypanosoma brucei gambiense* HAT live within 5-hour travel of a fixed health facility offering diagnosis and treatment for HAT
			**O1**: Weak laboratory capacity in rural health facilities	2. A combination of active and passive screening is required
			**O2**: Low attendance rates in some health facilities	2. Currently (2000–2012), half of HAT cases in the world are reported from passive screening
Lumbala et al.^13^	Analysis of routine HAT data reported to PNLTHA in DRC 2000–2012	1. National policy of health sector reform calls for integration	–	1. Over the period 2000–2012, the proportion of all HAT cases detected by passive screening in DRC remained stable around 50%.
				2. Active screening needs to be maintained, in areas of intense transmission
		2. The attendance rates in active screening remained fairly stable at 79% between 2000 and 2012.		
				3. Essential requirements for integrating HAT screening in the health center are : Functional and well-equipped health center, with adequate utilization rate, and trained and motivated staff
				4. Express fear about loss of quality when integrating HAT screening in primary healthcare services compared with CDTC
				5. Question whether integrated HAT screening is a mere consequence of disinvestment by international donors or a rational planned response to changing epidemiological context
Simarro et al.^51^	Analysis of global reported HAT data 2003–2012	–	**F**: Suggesting new diagnostic and therapeutic tools will facilitate future integrated approaches	–
Mitashi et al.^4^	Descriptive study of 43 primary healthcare centers in highly endemic areas in DRC	1. Integrated screening is part of the WHO policy on HAT elimination. “*Active case detection by mobile screening teams is to be conducted in all historic HAT foci until no more cases are detected at village level over a 5-year period. From that time onwards, screening should be shifted to passive, making use of existing general healthcare services.*”	**F1**: HAT well-known by staff	1. Integrated HAT screening in primary health centers requires not only specific HAT-related but also general strengthening of health services and increased utilization rates.
			**O1**: Very low attendance rate of health centers (median two patients per day in the week preceding survey)	
			**O2**. Poorly equipped centers. Only six of 9 CDTCs were fully equipped for HAT diagnosis and treatment. None of the 34 HCs was equipped for screening	
National policy on HAT control in DRC PNLTHA^54^	Policy paper	–	–	Flexible approach to integration is required, adapted to local context of endemic provinces, operational capacity and epidemiological context
Aksoy et al.^47^	Viewpoint	Cost Sustainability	–	In low endemicity areas, control strategies need to shift from active to passive screening in health centers
Kegels^49^	PhD thesis	–	**O:** A lot of internal and external resistance toward integration leads to a very slow process	–

CATT = card agglutination test for trypanosomiasis; CDTC = Centre de Diagnostic, Traitement et Contrôle; DRC = The Democratic Republic of the Congo; RDT = rapid diagnostic test; HAT = human African trypanosomiasis; PNLTHA = Programme National de Lutte contre la THA; PHC = primary healthcare; HC = health center. (Empty boxes means that the document you are viewing does not contain information related to the title of the column).

### Arguments for integrating HAT screening activities into first-line health services.

In the recent literature, four major arguments are evoked to justify the integration of HAT screening activities into the first-line health services:

#### Coverage (arguments related to coverage were found in the published or written literature after 1998).

First-line health services in HAT case detection in addition to active screening by mobile units has the potential to improve the coverage of the population at risk by casting a larger net (Table 2).^12^ There is definitely the need for such an extended network of first-line health centers offering HAT testing to at risk populations, a point convincingly made by Simarro et al.^12^ in their survey of the existing capacity for passive screening in endemic areas (Table 2). Exploiting data from a global HAT database managed by WHO, they identified 622 facilities in Central- and West Africa (more than 80% in DRC) involved in HAT case detection and management, although for 44% of these, their capabilities were limited to recognizing clinical suspects. If 56% of those facilities were able to carry out a serological test, only 49% were able to confirm and 39% to stage a HAT case. Stage 1 treatment was feasible in 80% of the centers, whereas treatment of stage 2 patients was only feasible in 36% of the structures. Forty-one percent of the total at-risk population lives within 1 hour of such a facility. This encouraging picture contrasts with a field survey carried out in 2015 by Mitashi et al. in a highly endemic region of DRC, where the low attendance rates of such primary health centers were identified as the most important weakness. This questions the fact whether effective coverage can be reached through passive screening in these highly endemic regions (Table 2).^4^ One explanation for this contrast is that the findings of Simarro et al.^12^ are heavily influenced by the CDTC centers, the dedicated HAT diagnosis and treatment centers (vertically) operated by the DRC national control program, and do not reflect the conditions in the general first-line health centers that were depicted by Mitashi et al.^4^ Simarro et al. mapped the existing capacity in fixed health facilities throughout Central and West Africa for HAT screening, diagnosis, and treatment. They obtained the data by interviewing the country directors of the HAT control programs. For example, for DRC, they reported 524 facilities in total. These included a few rural hospitals, but most facilities were CDTCs. This is clearly stated, e.g., in the annual report of the PNLTHA of 2012: “*Most health facilities are not able to perform sensitive tests for the diagnosis of HAT* (*CTC and mAECT*) *due to the lack of a 220V electricity source or lack of necessary equipment. Those who perform these techniques are for the most part the specialized structures of the HAT program* (*CDTC*) *which still depend on the PNLTHA or which have been integrated in the health district* (*CDTC integrated in general referral hospital or health centers of health district*)”.^38^ The authors point out this contribution of the integration of HAT into coverage^12,39,40^ at a time when there is great progress in research in diagnosis and treatment. Rapid diagnostic tests for use in peripheral structures and fexinidazole for oral administration will facilitate passive testing in health centers. The big challenge remains the underutilization of public primary healthcare services.

#### Cost.

Before 1998, Pepin et al.^41^ considered integrated HAT control as a less costly way of organizing sleeping sickness control (Table 1). And after 1998, Epco et al.^42^ had the same argument, and this is because of the low budget context (Table 2). In West Africa, active case-finding surveys are no longer cost-effective in most foci because of a dramatic decline of disease transmission (Table 2).^42^ Integrated sentinel surveillance in fixed structures has now become the alternative promoted by WHO in this region.

#### Quality of care.

In 1977, Mercenier was the first to state that the first-line health services have great potential for the control of HAT (Table 1),^43^ based on the features of primary care: a permanent service, close to the community, with high acceptability. He also demonstrated that about three quarters of all HAT patients detected in a district were or could have been detected at the primary care center level. He emphasized that they would have been detected earlier than the mobile team could have. However, this was true in an era before the inclusion of the sensitive card agglutination test for trypanosomiasis (CATT) test in the screening algorithm 1997 onward.^44^ Before that, patients were still screened essentially on clinical signs such as palpation of lymph nodes. Today, this is no longer true and many studies and routine data point out that the main disadvantage of passive detection is detecting people with a more advanced stage of the disease. However, this is partly due to inherent inefficiencies in the organization of case confirmation, as shown by Hasker et al.^45^ (Table 2), and could be considerably improved.

#### Sustainability.

History has repeatedly shown HAT control efforts should not be abandoned when the prevalence is low because it will re-emerge (Table 2).^7^ To achieve and sustain the global goal of HAT elimination as a public health problem, disease surveillance will need to be sustained in an efficient and cost-effective manner for many years to come, and more integrated approaches are frequently promoted in the literature as a way to achieve this (Table 2).^3,10,46^ Nevertheless, the functionality of basic health services for these integrated approaches should be taken into account despite the current availability of RDTs and the recent authorization for oral use of fexinidazole.

### Factors influencing the integration process and lessons learned from previous studies.

The existing literature points to numerous factors, which can either facilitate or negatively influence the integration of control into local health services. These are lessons learned to better integrate HAT activities. These factors can be categorized into three domains.

#### Factors related to the clinical evolution of HAT.

HAT is a relatively rare illness with none or few nonspecific symptoms in the first stage (Table 2),^42^ so infected people do not immediately seek care. It is typical for the case mix of patients detected in “passive mode,” that many are in an advanced stage, contrasting with those detected in active screening, who are predominantly at the first stage. This compromises individual prognosis but may also put communities at risk. In the past, fully operational first-line health services have missed out on an unfolding HAT resurgence (Table 2).^7^ A health seeking behavior survey showed that in passive screening, the patient's delay between the onset of symptoms and contact with a health center was short, but a significant delay was noted between screening and confirmation, which compromised the prognosis (Table 2).^45^ The combination of active and passive approaches may be a good alternative for managing all patients (symptomatic and asymptomatic). This opinion does not converge with the arguments of some authors who want to go from active screening to passive detection in the case of low prevalence.^47^

#### Factors related to the health system.

In 1977, the pre-CATT era, Mercenier analyzed a series of HAT cases in Kasongo district and pointed to the potential of the primary healthcare services for HAT control, based on the observation that a considerable proportion of HAT patients were detected on the basis of a spontaneous consultation in a primary healthcare center (Table 1).^43^ Afterward in 1989, Pépin et al.^43^ also argued, in the context of promoting primary healthcare, that in the DRC (then Zaire), the cost of integrated screening was lower and HAT cases detected in primary healthcare centers came to the more advanced (only 22% of stage 1 compared with 65% in mobile units). Primary healthcare centers could make a substantial contribution to HAT testing, meaning that 5 years after the start of the study, health centers detected 31% of all HAT cases in the health district. This experience has shown that active screening must be maintained and cannot be organized by the health center nurse like in the case of Nioki. The authors concluded that the essential conditions for the integration of HAT screening in the health center are: functional and well-equipped health center, with adequate utilization rate and trained and motivated staff (Table 1).^41^ De Brouwere and Pangu^8^ pointed out that HAT control should be preferably implemented in line with a policy of primary healthcare (Table 1). They discussed several possible models of integration. Integration, according to these authors is not a purpose in itself, and specialized (vertical) services are sometimes necessary depending on the local context. After 1998, several authors raised the challenges related to integration in the current state of primary care services. Several weaknesses precluding effective integration, such as the lack of skills of the staff, their lack of motivation and poor payment, low utilization rate of these health centers, lack of equipment and consumables, poor quality of diagnosis and treatment, shortage of drugs, and also the fact that staff considers this extra work rather negatively (Table 2).^4,42,46,48^ The capacity review by Mitashi et al. in 2015 on 43 primary healthcare centers in two highly endemic health zones (Mushie and Kwamouth) of the former Bandundu Province presented a sobering picture, given that those centers received on average one patient per day in the consultation, and, apart from skilled staff, also lacked the basic infrastructure, power supply, and cold chain to ensure HAT case detection (Table 2).^4^ This low attendance rate is a major hurdle and is compounded by the fact that in a context of elimination, each nurse at a center will very rarely encounter a HAT suspect. Whatever the epidemiological context, for the reasons mentioned earlier, the effective integration can take a long time to overcome internal and external resistance (Table 1).^49^ Some endemic foci may experience particular events such as in conflict situations with consequences such as lack of health infrastructure. This situation does not favor an integrated approach to HAT.^50^ On the other hand, the strengths of these primary care services are their permanence, the proximity they offer, and their multiple functions (Table 1).^43^

#### Factors related to the diagnostic and therapeutic tools.

As explained earlier, first-line health services do not have the capacity to carry out certain laboratory examinations such as confirmation and staging (Table 2).^4^ On the other hand, the RDTs, which are now available, can be easily used in the first-line health services and favor the integration of HAT detection (Table 2).^46,51,52^ Today, fexinidazole represents a real progress for treatment especially for people living in the most remote areas.^19^ Use of this orally available drug will address problems related to nursing and logistics administration of the NECT regimen in the second stage of HAT (Table 2).^53^

### Policy document review.

(All of the national or international policy documents considered in this review are those that have been made available by the PNLTHA-DRC and date back to years after 1998.)

Today, the debate on whether “to integrate or not” can be considered closed, as one of the recommendations of the WHO Expert Meeting in Geneva in 2013 includes passive HAT screening as one of the essential strategies in low endemic areas.^54^ In DRC, integration of disease control is a leading principle in the national health policy to avoid the fragmentation of the health system, as most vertical programs are backed by international donors. The DRC HAT subsector policy is the result of a participatory process of national, international, and Ministry of Health experts. The national HAT control policy in DRC stipulates since 2003 that the integration of the activities in local health services is an essential strategy to improve coverage.^55^ More recently, it takes into account the motions of the 66th World Health Assembly in 2013, which urged the member states to implement the interventions needed to achieve the goal of eliminating HAT as a public health problem by 2020. The 2011–2015 strategic plan pointed to some progress that was made. Training of physicians, nurses, and laboratory workers was organized in some endemic health zones; the CATT test was adopted as a marker in the blood transfusion services; and the proportion of the screened patients in the health centers and the general hospital of reference went from 15% to 18% from 2007 to 2009.^55^

## DISCUSSION

Although our review shows the many pitfalls and complexity of integrating HAT control, lessons learned from previous integration attempts constitute essential elements to guide any integration process in the primary healthcare today. Our scoping review starts toward the end of the 1970s, when the debate became more pragmatically oriented than in the immediate post-colonial era. Until now, there is no well-established consensus on what the ideal model for HAT detection and management should be, and there is probably no one-size-fits-all answer. Integrated approaches of HAT screening offered in fixed health centers have potential in terms of acceptability, access, and continuity of care but also show the downside of diagnosing patients much later in an advanced stage 2 with worse prognosis. Integrated disease control presents both opportunities and threats for the vertical program as well as for the first-line health services.^56^ Instead of opposing both approaches, the answer seems more to lie in finding the right mix of both horizontal and vertical approach depending on context. The primary challenge then becomes how to optimize the interface between both, the specialized approach of the vertical program with the horizontal one of the local health services. Today, authors discuss the prerequisites of successful integration in terms of a certain minimum level of functionality of the first-line health services and a prevalence of HAT that is not too low.^33^

In DRC, the encouraging downward trend in number of HAT cases has led to diminishing efficiency of the active screening. The decreasing HAT incidence is unfortunately often correlated with a decreasing uptake of screening campaigns by the population. Fewer cases in villages means that people are less motivated to attend the lengthy screening sessions because they do not like to waste time waiting in queues for a problem that has almost disappeared from their daily life. In Equateur province in DRC, the epicenter of the 1998 epidemic, the community uptake in active screening sessions has now dwindled to 42% in 2015.^57^ Even when better uptake is achieved, the cost per HAT case detected and treated inevitably soars with decreasing screening yield. The frequency of confirmed HAT cases among the population screened in Equateur-Nord in 2015 was as low as 1.5 per 10,000, with 19 cases detected in a population screened of 125,810. The costs were estimated at 1.5 EUR per person screened, whereas the average cost per HAT-confirmed case was more than 2,000 EUR (PNLTHA, unpublished data). Based on the 2015 data, the opportunity cost of active screening for HAT becomes an important consideration.

We are aware that evolving technology may change the parameters of this debate quickly, as what was not feasible in a primary healthcare center in the past, may become feasible in the future because of simpler and more appropriate technology. The first experiences of integration of diagnosis and treatment of HAT in the general health services date back to the late 1970s,^41,43^ when the complexity of the diagnostic procedures requiring parasitological confirmation by microscopy and a lumbar puncture for the determination of the stage hindered a real integration.^58^ So far, there is tremendous progress being made. The oral treatment (fexinidazole) has been approved for use and constitutes a real technological advance that will determine to a large extent the effectiveness of integrated approaches. The simplest oral regimen will be acoziborole, if it completes satisfactorily ongoing clinical trials. Decision analysis has to examine whether the current algorithm can change from 1). screen by RDT, 2). confirm parasitologically, and 3). treat to 1). test by RDT and 2). treat if RDT positive. These technological breakthroughs and a substantial reduction in the number of reported HAT cases each year^2^ not only changed the prospects from control to elimination but also the perspectives for integrated approaches, as the new tools are more appropriate for use in primary care settings.

The desire for service integration in an elimination context (today) goes further than what has been undertaken in HAT ever before, either because programs are expected to implement passive screening in more challenging contexts and/or because programs are expected to rely on passive screening as the primary strategy for detecting cases.

In general, from our scoping review, we can formulate the following recommendations for the process of integration. Strengthening the primary healthcare services is a prerequisite to obtain an acceptable integration of HAT control activities in endemic areas. Integration of HAT services into primary healthcare services is not an easy process because—so far—the package of HAT care (screening, confirmation, and treatment) is complex and transfer of responsibility does not make sense if the horizontal services are poorly trained and ill-equipped. The integration of HAT control should, therefore, lead to the active participation of a strengthened primary healthcare service capable of implementing HAT surveillance and control activities.^10^ The training of caregivers, the supply of basic consumables, the construction and rehabilitation of infrastructures, technical support, and supervision are key recommendations.^43,45,48^ Innovation toward more appropriate tools will pave the way. The current management of HAT still presents a high technical complexity, but at a lower level compared with other diseases such as HIV and TB (HAT is a short duration treatment) that have successfully integrated some of their services. The advent of RDTs and the approval of fexinidazole as the first oral treatment for HAT have opened new perspectives and will certainly change the situation in the near future.^2,19^ In general, this domain requires a context-sensitive “diagonal” approach. Teamwork between the specialized vertical program and the first-line health services is encouraged. So, lessons for the elimination era are that integrated approaches have potential, but essential requirements need to be met, and a certain degree of “vertical” technical supervision is required to assure quality of the work. In conflict zones, logistical and medical capacity has to be balanced with security considerations; community networks and international coordination should be maintained, and this may require vertical approaches.^50^ The “hybrid” CDTC structure in DRC seems a successful model and an example of a diagonal approach, as many of these centers are physically located within the premises of the general hospital.

## CONCLUSION

Integration of HAT control into primary healthcare services is possible but still requires prior investments in terms of materials, equipment, and training. An active approach has to be maintained as long as primary healthcare services are not functioning properly and are underutilized. Because of the complexity of the diagnosis of sleeping sickness, a two-level identification system is still required: screening with an RDT followed by parasitological confirmation. As a result, a referral and counter-referral system for patients will need to be better organized at the operational level of care. The current availability of safe oral therapy (fexinidazole) and the current use of RDTs at the peripheral health center will move elimination of HAT forward. To maintain the skills of peripheral health workers on the disease in the context of low prevalence, continuing education, and close formative supervision should be organized in endemic health areas. The integration of HAT at such a time requires resources, and donor support has to be maintained. Currently, this integration is to be encouraged as it is likely to accelerate progress toward universal health coverage while contributing to the broader sustainable development goals set for 2030.
